# Extensive Osmotic Demyelination Syndrome With a Concomitant Cytotoxic Lesion of the Corpus Callosum in Hyperemesis Gravidarum: A Case Report

**DOI:** 10.7759/cureus.108825

**Published:** 2026-05-14

**Authors:** Salma Abouchiba, Nizar El Bouardi, Hajar Ouazzani, Ismail Chaouche, Amal Akammar, Meriem Haloua, Badreddine Alami, Y. Lamrani, Meryem Boubbou, Mustapha Maaroufi

**Affiliations:** 1 Radiology Department, Hassan II University Hospital, Sidi Mohamed Ben Abdellah University, Fez, MAR; 2 Mother and Child Radiology Department, Hassan II University Hospital, Sidi Mohamed Ben Abdellah University, Fez, MAR

**Keywords:** cytotoxic lesion of the corpus callosum, extrapontine myelinolysis (epm), hyperemesis gravidarum (hg), osmotic demyelination syndrome (ods), severe hypernatremia

## Abstract

Osmotic demyelination syndrome (ODS) is a rare neurological disorder classically associated with rapid correction of hyponatremia but increasingly recognized in the setting of complex osmotic disturbances. Its radiological spectrum includes central pontine and extrapontine myelinolysis, with variable clinical and imaging presentations. The coexistence of cytotoxic lesions of the corpus callosum (CLOCCs) in this context remains exceptional and poorly understood. We report the case of a 35-year-old pregnant woman presenting with severe hyperemesis gravidarum complicated by profound electrolyte imbalance and marked fluctuations in serum sodium levels. Initial brain magnetic resonance imaging (MRI) was unremarkable despite progressive neurological deterioration. A follow-up MRI performed two weeks later revealed extensive symmetric T2-weighted/fluid-attenuated inversion recovery (FLAIR) hyperintensities involving the pons, basal ganglia, thalami, hippocampi, internal and external capsules, and bilateral cerebellar hemispheres. Diffusion-weighted imaging (DWI) demonstrated heterogeneous findings, with restricted diffusion limited to the capsular regions. Additionally, a focal lesion in the corpus callosum with marked diffusion restriction was identified, consistent with a CLOCC. Mild contrast enhancement was observed within the pontine lesion. Extensive pontine and extrapontine involvement combined with CLOCC represents a rare but significant manifestation of osmotic brain injury. Recognition of this association and awareness of delayed imaging findings are essential for accurate diagnosis and management.

## Introduction

Osmotic demyelination syndrome (ODS) is an uncommon but potentially devastating neurological disorder classically associated with rapid osmotic shifts, most frequently following overly rapid correction of chronic hyponatremia [1,2]. It encompasses both central pontine myelinolysis and extrapontine myelinolysis, reflecting the selective vulnerability of myelin-rich regions of the central nervous system to osmotic stress [1,3,4]. Although the pons is the most commonly involved structure, extrapontine lesions involving the basal ganglia, thalami, cerebellum, and subcortical white matter are increasingly recognized [3,5,6], particularly with the widespread use of magnetic resonance imaging (MRI) [7].

While ODS has historically been linked to correction of hyponatremia, it is now well established that a variety of hyperosmolar states, including hypernatremia, severe dehydration, malnutrition, hyperosmolar hyperglycemic state (HHS), and systemic illness, may also precipitate the condition [8,9]. Pregnancy-related disorders, particularly hyperemesis gravidarum, represent a rare but clinically relevant context in which profound electrolyte disturbances and rapid osmotic shifts may occur [10,11]. In such settings, the combination of dehydration, nutritional deficiency, and therapeutic interventions may synergistically contribute to the development of osmotic demyelination [12,13].

MRI plays a central role in the diagnosis of ODS; however, imaging findings may be delayed, and early examinations can be unremarkable despite significant clinical deterioration [6,7]. Furthermore, atypical imaging features and unusual associations may be encountered, reflecting the complexity of the underlying pathophysiological mechanisms [10].

We report the case of a 35-year-old pregnant woman who developed extensive ODS in the setting of hyperemesis gravidarum and severe electrolyte imbalance. The case is notable for the unusually widespread distribution of lesions involving both pontine and extrapontine structures, heterogeneous diffusion characteristics, and the concomitant occurrence of a cytotoxic lesion of the corpus callosum (CLOCC), suggesting overlapping mechanisms of osmotic and metabolic brain injury.

## Case presentation

A 35-year-old pregnant woman with no significant past medical history was admitted for management of severe hyperemesis gravidarum during early pregnancy. Her initial clinical course was characterized by persistent vomiting, markedly reduced oral intake, and progressive dehydration. During hospitalization, she developed progressive alteration of mental status, which rapidly evolved into a severe disorder of consciousness, prompting transfer to the intensive care unit.

Biochemical investigations revealed profound electrolyte disturbances, most notably significant fluctuations in serum sodium levels. Serum sodium increased from 146 mmol/L to a peak of 180 mmol/L over a short period, followed by partial correction to values ranging between 156 and 163 mmol/L. These rapid osmotic shifts occurred in the context of ongoing metabolic derangement and therapeutic interventions. Concomitantly, the patient exhibited persistent hypokalemia, with potassium levels as low as 2.7 mmol/L, further contributing to cellular vulnerability to osmotic stress (Table 1).

**Table 1:** Evolution of key biochemical parameters during hospitalization

Chronicity	Sodium (mmol/L)	Potassium (mmol/L)	Urea (g/L)	Creatinine (mg/dL)
First day of admission	146	4.0	0.44	0.9
Six days later	180	2.7	0.51	0.7
Nine days later	156	3.9	0.33	0.7
14 days later	163	2.9	0.21	0.7
Reference values	136-145	3.5-5.0	0.15-0.45	0.5-1.1

Upon admission to the intensive care unit, the patient presented with severe impairment of consciousness, with a Glasgow Coma Scale score of 6, without meaningful interaction with her surroundings, although brainstem reflexes were preserved. Her clinical course was further complicated by an infectious syndrome characterized by febrile episodes and elevated inflammatory markers, as well as nutritional deficiency requiring enteral feeding. Supportive management included respiratory support, electrolyte correction, antimicrobial therapy, and nutritional supplementation.

Imaging findings

An initial brain MRI performed approximately 48 hours after admission, prior to the onset of impaired consciousness, was unremarkable, with no abnormal signal intensity on T2-weighted or fluid-attenuated inversion recovery (FLAIR) sequences, no diffusion restriction, and no pathological enhancement.

**Figure 1:**
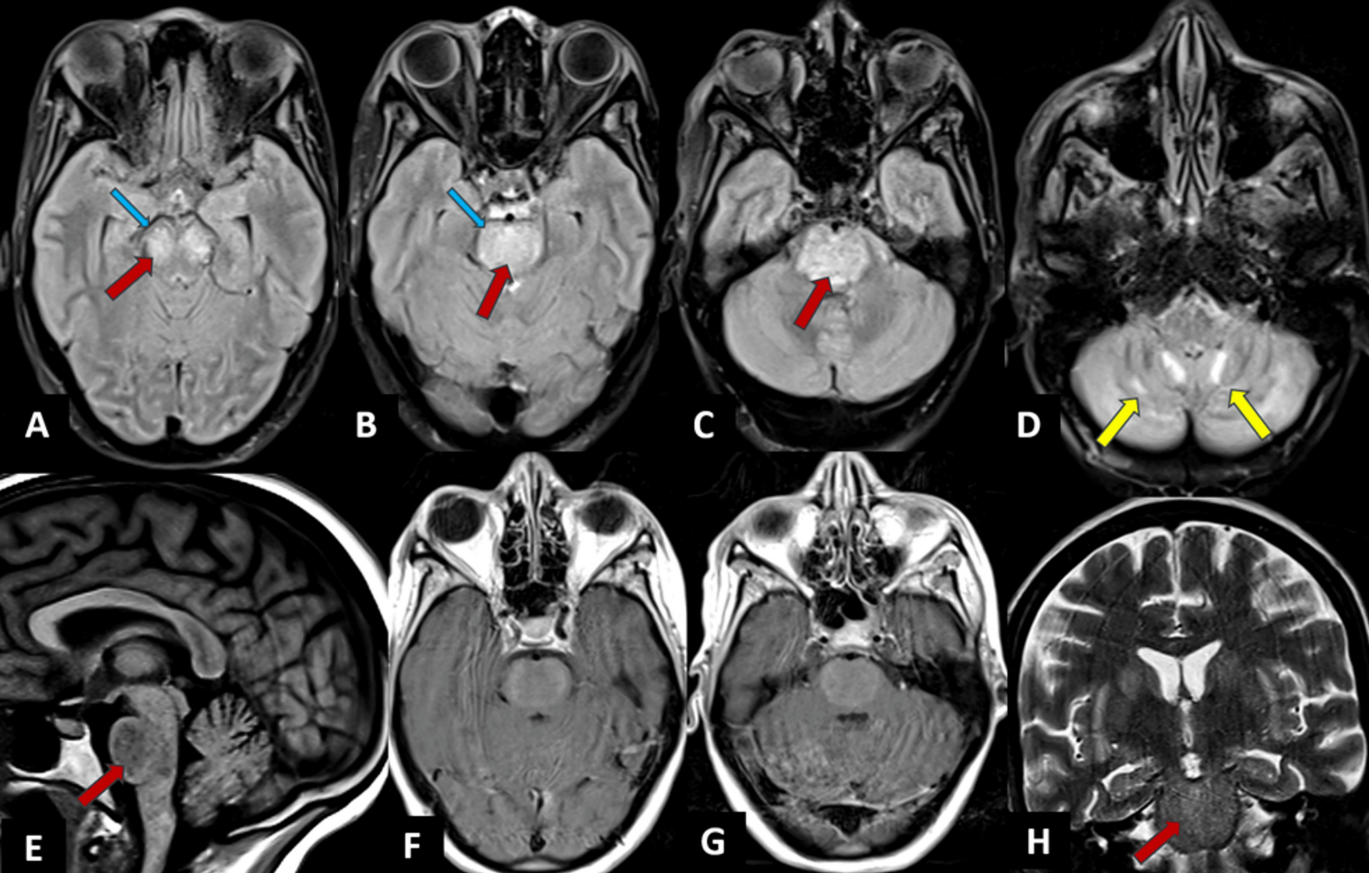
Pontine and extrapontine involvement in ODS Axial FLAIR images (A-C), sagittal T1-weighted image (E), and coronal T2-weighted image (H) demonstrate a symmetric central pontine lesion, appearing hyperintense on T2-weighted and FLAIR images and hypointense on T1-weighted imaging (red arrows), with relative sparing of the peripheral fibers (blue arrows). Post-contrast images (F, G) demonstrate faint patchy enhancement within the lesion. Axial FLAIR image (D) demonstrates bilateral extrapontine cerebellar lesions (yellow arrows). FLAIR: fluid-attenuated inversion recovery; ODS: osmotic demyelination syndrome

A follow-up MRI performed 14 days later demonstrated extensive and symmetric signal abnormalities involving both pontine and extrapontine structures. At the level of the pons, a central lesion appeared hyperintense on T2-weighted and FLAIR images and hypointense on T1-weighted images. The lesion showed faint patchy enhancement following contrast administration, without associated diffusion restriction, a pattern consistent with subacute central pontine myelinolysis and suggestive of blood-brain barrier disruption (Figure 1).

**Figure 2:**
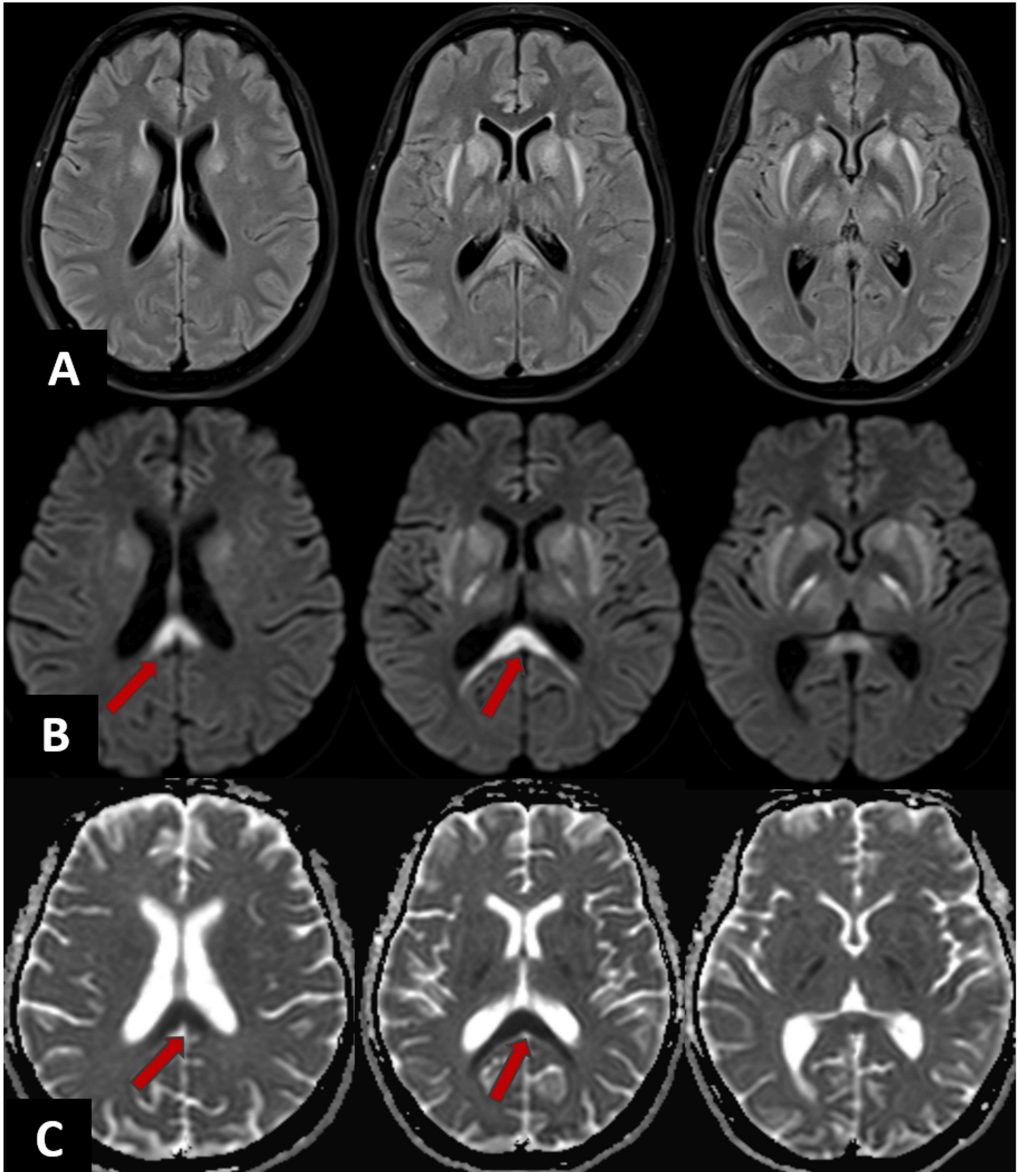
Extrapontine involvement and associated CLOCC Axial FLAIR image (A) demonstrates symmetric hyperintensities involving the caudate nuclei, putamina, thalami, internal and external capsules, hippocampi, and cerebellar hemispheres. Axial DWI (B) and ADC map (C) demonstrate mild diffusion restriction limited to the capsular regions. A focal splenial lesion demonstrates marked diffusion restriction on DWI with corresponding low ADC values, consistent with a CLOCC (red arrows). CLOCC: cytotoxic lesion of the corpus callosum; DWI: diffusion-weighted imaging; ADC: apparent diffusion coefficient; FLAIR: fluid-attenuated inversion recovery

In addition to pontine involvement, multiple extrapontine regions were affected, including the caudate nuclei, putamina, thalami, internal and external capsules, hippocampi, and bilateral cerebellar hemispheres. These lesions were symmetrically distributed and appeared hyperintense on T2-weighted and FLAIR sequences, without significant mass effect or hemorrhagic component. Diffusion-weighted imaging (DWI) revealed mild diffusion restriction limited to the internal and external capsules, whereas the remaining lesions, including those involving the pons, basal ganglia, thalami, and cerebellum, did not demonstrate restricted diffusion. This heterogeneous diffusion pattern suggests variable temporal evolution of the lesions, with capsular involvement likely corresponding to more acute injury, while the other regions may represent a more advanced, subacute stage of demyelination (Figure 2).

Notably, a focal lesion was identified within the corpus callosum, characterized by marked diffusion restriction, hyperintensity on DWI, and corresponding low apparent diffusion coefficient (ADC) values, highly suggestive of a CLOCC.

Overall, the imaging findings were consistent with extensive ODS involving both central pontine and widespread extrapontine regions, associated with a concomitant CLOCC and exhibiting heterogeneous temporal and radiological characteristics.

Patient outcome 

Following the acute phase, the patient remained in the intensive care unit and required tracheostomy for airway management. She was maintained on parenteral nutrition because of the absence of oral intake. Her level of consciousness remained severely impaired, with a Glasgow Coma Scale score persistently at 7, and she exhibited no meaningful responsiveness to external stimuli throughout the follow-up period.

## Discussion

ODS represents a rare but potentially devastating neurological complication resulting from rapid osmotic shifts within the central nervous system. Initially described in the context of overly rapid correction of chronic hyponatremia, ODS is now recognized as a broader entity encompassing both central pontine and extrapontine myelinolysis, reflecting a continuum of demyelinating injury affecting selectively vulnerable brain regions [1]. Increasing evidence suggests that ODS may arise not only from correction of hyponatremia but also from hypernatremia and large fluctuations in serum osmolality, emphasizing that the rate and magnitude of osmotic change, rather than the absolute sodium value alone, are the critical determinants of injury [1,2].

The underlying pathophysiological mechanisms are closely related to the adaptive response of brain cells to chronic osmotic imbalance. In hyponatremic states, neurons and glial cells reduce intracellular osmolytes to maintain osmotic equilibrium and prevent cerebral edema. Rapid correction disrupts this equilibrium, leading to cellular dehydration, astrocytic injury, and blood-brain barrier disruption, ultimately resulting in oligodendrocyte damage and noninflammatory demyelination [1,2]. Additional factors such as hypokalemia, malnutrition, and systemic illness further impair cellular resilience and amplify susceptibility to osmotic injury, likely contributing to the severity and extent of lesions observed in our patient [3].

Hyperemesis gravidarum represents a particularly complex metabolic context in which multiple electrolyte disturbances may develop simultaneously and interact to amplify the risk of osmotic brain injury. Beyond the well-recognized association with hyponatremia, severe hyperemesis gravidarum can precipitate hypernatremia through profound dehydration and inadequate fluid replacement, as illustrated in our case. Additional electrolyte derangements frequently encountered in this setting include hypokalemia, hypomagnesemia, and hypochloremic metabolic alkalosis, all of which further compromise cellular homeostasis and increase vulnerability to osmotic stress [4]. While hyponatremia-associated ODS has been more extensively reported, hypernatremia-induced ODS, although less common, appears to operate through analogous mechanisms. In hypernatremic states, brain cells accumulate organic osmolytes to counteract cellular shrinkage, and subsequent osmotic correction, even if partial, may induce relative intracellular hypo-osmolarity, triggering demyelinating injury through mechanisms similar to those observed following correction of hyponatremia [1,2]. The severity of ODS in our patient likely reflects the combined effects of extreme hypernatremia, hypokalemia, malnutrition, and the systemic stress associated with prolonged critical illness.

Clinically, ODS is characterized by a typically biphasic course, with an initial phase of encephalopathy related to metabolic disturbances, followed by delayed neurological deterioration corresponding to demyelination. The clinical spectrum is heterogeneous, ranging from mild cognitive impairment to severe neurological deficits, including dysarthria, dysphagia, movement disorders, spastic quadriparesis, and disorders of consciousness [3]. Severe presentations, particularly those associated with extensive extrapontine involvement, are frequently correlated with poorer neurological outcomes, highlighting the prognostic significance of lesion distribution [3,5].

MRI is the cornerstone of diagnosis; however, its sensitivity in the early phase remains limited. A well-documented temporal dissociation exists between clinical onset and radiological abnormalities, with initial MRI examinations often appearing normal despite ongoing neurological deterioration [6,7]. This delay reflects the time required for structural myelin damage to become detectable on conventional imaging sequences and was clearly illustrated in our case, in which the initial MRI, performed early after admission and prior to the onset of impaired consciousness, was unremarkable despite ongoing metabolic disturbances and clinical deterioration. This finding underscores the importance of repeat imaging when clinical suspicion remains high.

Typical MRI findings of ODS include symmetric T2-weighted and FLAIR hyperintensities involving the central pons and extrapontine regions, most commonly the basal ganglia, thalami, and cerebellum [6,7]. The characteristic “trident-shaped” pontine appearance reflects selective vulnerability and relative sparing of the corticospinal tracts [6]. In more severe cases, however, a wider distribution of lesions may be observed, including involvement of the hippocampi, internal and external capsules, and cerebellar hemispheres, as demonstrated in our patient. Such extensive involvement likely reflects the magnitude and rapidity of osmotic stress and supports the concept of ODS as a diffuse vulnerability of myelin-rich structures under extreme metabolic conditions [7].

DWI plays a pivotal role in the early detection and characterization of lesions. Diffusion restriction may be observed in the acute phase, reflecting cytotoxic edema, but is not uniformly present and may vary depending on the stage of lesion evolution [7,8].

The heterogeneous diffusion pattern observed in our case, with restriction confined to the capsular regions, may suggest asynchronous lesion development, with coexistence of acute and subacute stages across different anatomical structures. This observation underscores the dynamic nature of ODS and highlights the importance of integrating diffusion findings into temporal staging.

The diffusion restriction confined to the internal and external capsules may also reflect corticospinal tract involvement, with early axonal injury and tract degeneration producing cytotoxic edema. Similar diffusion patterns have been described in ischemic injury, in which Wallerian degeneration of the corticospinal tracts manifests as transient DWI hyperintensity, supporting a tract-oriented mechanism for the focal capsular restriction [9].

Contrast enhancement is not a constant feature but may occur during subacute stages, reflecting blood-brain barrier disruption and ongoing tissue injury [5]. The faint pontine enhancement observed in our patient is consistent with this phase and further supports the temporal evolution of the lesions.

A particularly noteworthy aspect of this case is the coexistence of a CLOCC, characterized by marked diffusion restriction. CLOCCs, especially those involving the splenium, are increasingly recognized in association with metabolic disturbances, infections, and systemic stress, and are thought to result from transient cytotoxic edema mediated by excitotoxic mechanisms [9,10]. Unlike ODS, which primarily affects oligodendrocytes and myelin, CLOCCs reflect reversible neuronal and astrocytic dysfunction. The coexistence of a CLOCC with widespread osmotic demyelination lesions in our patient suggests the presence of overlapping but distinct pathophysiological processes, involving both osmotic injury to oligodendrocytes and transient cytotoxic edema affecting neuronal and glial elements, two mechanisms simultaneously activated by the extreme metabolic stress sustained by this patient.

The coexistence of CLOCCs and ODS, although rarely reported, suggests the simultaneous activation of distinct but overlapping pathophysiological processes. Severe metabolic stress may induce both osmotic demyelination and excitotoxic edema, affecting different cellular compartments within the brain. Recent reports have described similar associations, reinforcing the hypothesis that ODS and CLOCCs may represent complementary manifestations within a broader spectrum of metabolic brain injury [11]. This dual mechanism provides a compelling explanation for the mixed imaging pattern observed in our patient and constitutes a key element of the originality of this case.

Management of ODS remains largely supportive, as no specific therapy has been proven to reverse established demyelination. Prevention through careful correction of electrolyte disturbances remains the cornerstone of management [12]. Prognosis is variable and depends on the extent of lesions and clinical severity. While partial recovery is possible, severe cases with extensive extrapontine involvement and impaired consciousness are associated with a higher risk of persistent neurological deficits and long-term disability [12,13].

## Conclusions

This case illustrates an unusually extensive form of ODS associated with hypernatremia and marked osmotic fluctuations in the setting of hyperemesis gravidarum. The widespread distribution of lesions, heterogeneous diffusion characteristics, and coexistence of a CLOCC highlight the complexity of osmotic brain injury and emphasize the importance of recognizing overlapping radiological patterns. The severity of the clinical outcome further underscores the devastating potential of hypernatremia-induced ODS, particularly when compounded by concomitant electrolyte derangements, malnutrition, and the systemic stress inherent to severe hyperemesis gravidarum. From a neuroradiological perspective, this case underscores the need for repeat imaging in clinically deteriorating patients and provides insight into the multifactorial nature of osmotic and metabolic brain injury.
